# Contributions of Understory and/or Overstory Vegetations to Soil Microbial PLFA and Nematode Diversities in Eucalyptus Monocultures

**DOI:** 10.1371/journal.pone.0085513

**Published:** 2014-01-10

**Authors:** Jie Zhao, Songze Wan, Chenlu Zhang, Zhanfeng Liu, Lixia Zhou, Shenglei Fu

**Affiliations:** 1 Key Laboratory of Vegetation Restoration and Management of Degraded Ecosystems, South China Botanical Garden, Chinese Academy of Sciences, Guangzhou, China; 2 State Key Laboratory of Cotton Biology, Key Laboratory of Plant Stress Biology, College of Life Sciences, Henan University, Kaifeng, China; 3 Key Laboratory of Agro-Ecological Processes in Subtropical Region, Institute of Subtropical Agriculture, Chinese Academy of Sciences, Changsha, China; Tennessee State University, United States of America

## Abstract

Ecological interactions between aboveground and belowground biodiversity have received many attentions in the recent decades. Although soil biodiversity declined with the decrease of plant diversity, many previous studies found plant species identities were more important than plant diversity in controlling soil biodiversity. This study focused on the responses of soil biodiversity to the altering of plant functional groups, namely overstory and understory vegetations, rather than plant diversity gradient. We conducted an experiment by removing overstory and/or understory vegetation to compare their effects on soil microbial phospholipid fatty acid (PLFA) and nematode diversities in eucalyptus monocultures. Our results indicated that both overstory and understory vegetations could affect soil microbial PLFA and nematode diversities, which manifested as the decrease in Shannon–Wiener diversity index (*H′*) and Pielou evenness index (J) and the increase in Simpson dominance index (λ) after vegetation removal. Soil microclimate change explained part of variance of soil biodiversity indices. Both overstory and understory vegetations positively correlated with soil microbial PLFA and nematode diversities. In addition, the alteration of soil biodiversity might be due to a mixing effect of bottom-up control and soil microclimate change after vegetation removal in the studied plantations. Given the studied ecosystem is common in humid subtropical and tropical region of the world, our findings might have great potential to extrapolate to large scales and could be conducive to ecosystem management and service.

## Introduction

Biodiversity plays a crucial role in ecosystem function and processes not only because of its importance in production of food, fiber and fuel, but also because of its roles in ground water replenishment, flooding controls, prevention of soil erosion and ecological invasion, influences on restoration succession, and so on [1], [2]. Although the relationship between biodiversity and ecosystem functions and processes has been the focuses of quite a few studies over the last two decades, much controversy still exists [3]. The impact of plant diversity on ecosystem functions and processes has been explored mainly in grasslands (e.g. [4], [5]); and primary productivity is frequently used to assess the ecosystem stability [4]. In general, ecosystems with high biodiversity level are more stable, sustainable, reliable and predictable [1], [4], [5], [6]. By contrast, several studies reported no significant effect of plant diversity on plant productivity [7], [8].

Soil contains the most diverse communities of organisms and soil biodiversity provides a number of ecosystem services [2], [9]. Ecological interactions between aboveground and belowground biodiversity have received many attentions in the recent decades [10]. In one hand, plants play a key role in determining soil biota because plants are the main food sources (i.e. leaf litter, dead roots and root exudates) to soil biota, which may exert a bottom-up control on soil organisms. In another hand, soil biota may affect aboveground plant diversity and productivity through influencing nutrient cycling and distribution, and damaging plants by parasites, pathogens and herbivores [11], [12].

Plant functional groups (PFG) or plant functional types (PFT) are assemblages of species with similar roles in ecosystem processes by responding in similar ways to multiple environmental factors [13]. They are necessary devices for reducing the complexity of plant community and often uncharted characteristics of species diversity in function and structure when attempting to project the nature and function of species assemblages into future environments [14]. However, due to the diverse aims of different researches, diverse plant functional groups are chosen to simplify the plant community, i.e. legumes, forbs/herbs, C3/C4-plants, overstory vegetation, and understory vegetation.

Vegetation removal has been frequently used to explore how specific plant species or functional groups affect ecosystem processes and functions. Overstory removal (timber harvest) has been a common forest management practice which also provides opportunities for soil ecologists to study relationships between overstory tree species and soil biota. In general, overstory removal suppresses soil biota such as bacteria, fungus, nematodes, and microarthropods in the long run [15], [16], [17]. However, there are studies which reported overstory removal had slight or non-significant impacts on soil biota, especially in the short term [18], [19]. To date, less attention has been paid to understory vegetation in terms of their impacts on soil biota, probably because the biomass and economic value of the understory are less than overstory tree species. Understory vegetation is an important component in many forest ecosystems worldwide [20], [21] and drives ecological processes and functions, such as biodiversity, stand productivity, tree-seedling regeneration, forest succession, litter decomposition, soil nutrient cycling and soil water conservation [19], [22], [23], [24]. However, how understory vegetation affects the soil biodiversity has never been studied before.

In the present study, we monitored the soil microbial phospholipid fatty acid (PLFA) and nematode diversity after overstory and/or understory removal in eucalyptus monocultures in South China. The eucalyptus species is *Eucalyptus urophylla*, which is a fast-grow species and has been widely planted for reforestation in South China [19]. The understory vegetation was dominated by *Dicranopteris dichotoma*, a common fern species in humid subtropical and tropical region [20]. Other common understory species included *Miscanthus sinensis* and *Rhodomyrtus tomentosa*. Thus, the diversity of overstory and understory vegetations is not high in the studied ecosystems. Therefore, we did not calculate the plant diversity. In this experiment, overstory and understory vegetations were removed separately or in combination to assess their impacts on soil microbial PLFA profile and nematode community. Both *E. urophylla* and *D. dichotoma* are high in biomass and primary productivity [20], [25]. Previous study, conducted at the same site as this study, reported that understory removal showed stronger effects on soil microbial community structure, and abundance of nematode and microarthropod than overstory removal [20]. Therefore, the hypothesis of this study was that the effects of understory removal on soil microbial and nematode diversity were greater than overstory removal. The primary goal of this study was to evaluate the contributions of the understory and/or overstory vegetation to soil microbial and nematode diversity. Our analyses also intend to address the question of which factors (i.e. resource and soil microclimate) affected soil biodiversity after altering of aboveground vegetations.

## Materials and Methods

### Site Description

This study was conducted at the Heshan Hilly Land Interdisciplinary Experimental Station (112°50′E, 22°34′N), property of the Chinese Academy of Sciences (CAS) in Guangdong Province of China. The climate is subtropical monsoon with a distinct wet and dry Season. The mean annual temperature and precipitation are 21.7°C and 1,700 mm, respectively. The soil is an acrisol [26]. Vegetation at the experimental site consisted of three (replications) 4-year-old *E. urophylla* plantations. The seedlings were planted with a spacing of 3×2 m.

### Experimental Design

Our experiment was conducted in three replicated plots of 4-year-old *E. urophylla* monoculture plantations. In September 2009, we established an experiment with a split-plot design. Within each plantation, main plots (15×15-m) were overstory trees treatment (present or removed) and subplots were understory treatment (present or removed). In total, there were four treatments in each plantation, namely, control with intact overstory and understory (CK), understory removal (UR), overstory tree removal (TR) and all-plant removal (PR). The trees were cut with an electrical saw leaving 5 cm stumps aboveground. All woody debris was removed from the plots immediately after vegetation was removed. The shoots of all understory plants were removed manually with the aid of a machete. Trenches were created around each main-plot and subplot to prevent the interactions of the roots from other subplots and to prevent the exchange of nutrients among subplots. Germination of the stumps (TR plots) and understory vegetation (UR and PR subplots) were monthly removed.

### Soil Sampling and Analysis

Soils were sampled on 20 September, 1 October and 6 November 2009, 11 March, 15 June and 14 October 2010, corresponding to 10 days before and 7, 37, 160, 256, 376 days after treatments were implemented. Soil cores (2.5 cm in diameter, 5 cm in length) were taken at 0–5 cm and 5–10 cm depths from eight locations selected randomly in each subplot within each plantation. Eight cores of the same depth from each subplot were combined to form one composite sample; there were three replicate samples for each treatment. The surface litter was removed carefully when soil sample was taken.

Soil water content (SWC %, g of water per 100 g dry soil) was measured by oven-drying for 48 h at 105°C. Soil temperature was recorded every 2 h with the DS1922L temperature logger iButtons (Dallas Semiconductor Corp., Dallas, TX) from September 2009 to October 2010. Soil microbial PLFAs were analyzed according to [27]. Briefly, the soil was extracted in a one-phase extraction mixture containing chloroform:methanol:phosphate buffer (1∶2∶0.8 v/v/v), with the amount of phosphate buffer corrected to account for existing soil water content mixture of chloroform:methanol:citrate buffer (1∶2∶0.8 v/v/v). After extraction the phospholipids were separated from neutral lipids and glycolipids on solid phase extraction columns, 0.50 g Si (Supelco, Inc., Bellefonte, Penn). Polar lipids were eluted and were then subjected to mild alkaline methanolysis. Resulting fatty acid methyl esters (FAMEs) were separated, quantified, and identified using capillary gas chromatography (GC). Qualitative and quantitative fatty acid analyses were performed using an Agilent 6890 gas chromatograph (Agilent Technologies, Palo Alto, CA, USA) and the MIDI Sherlock Microbial Identification System (MIDI Inc., Newark, DE, USA). Concentrations of each PLFA were calculated relative to 19∶0 internal standard concentrations. The following PLFAs were considered to be of soil microbial origin: i15∶0, a15∶0, 15∶0, i16∶0, 16∶1ω7, i17∶0, a17∶0, 17∶0, cy17∶0, cy19∶0 18∶2ω6,9, 16∶1ω5, 18∶1ω7, and 18∶1ω9 [28], [29], [30].

Nematodes were extracted from 50 g of fresh soil using the Baermann funnel method. After fixation in 4% formalin solution, nematodes were counted with a differential interference contrast (DIC) microscope (ECLIPSE 80 i, Nikon), and the first 100 individuals encountered were identified to genus level. All nematodes were identified to genus level when the sample contained fewer than 100 individuals.

### Statistical Analysis

Shannon–Wiener diversity index, Pielou evenness index, Margalef richness index, and Simpson dominance index were employed to assess the soil microbial PLFA and nematode diversity.
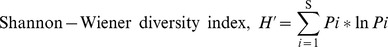
(1)

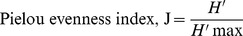
(2)

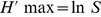
(3)

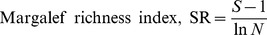
(4)

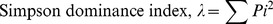
(5)


Where ‘*Pi*’ is the proportion of the individuals of “*i*th” group in the community; ‘*S*’ is the total number of microbial PLFAs or nematode genera in the community; and ‘*N*’ is the amount of microbial PLFAs or number of total nematodes in the community.

Repeated-measure ANOVA was employed to determine the time effect and treatment effect through the whole experimental period with TR and UR as between-subjects factors. To address the relationship between soil microclimates (i.e. soil water content and soil temperature) and soil biodiversity, linear and quadratic regression analysis were performed. Repeated-measure ANOVA and regression model were performed using SPSS software (SPSS Inc., Chicago, IL). Statistical significance was determined at *p*<0.05.

## Results

### Microbial PLFA Diversity

**Figure 1:**
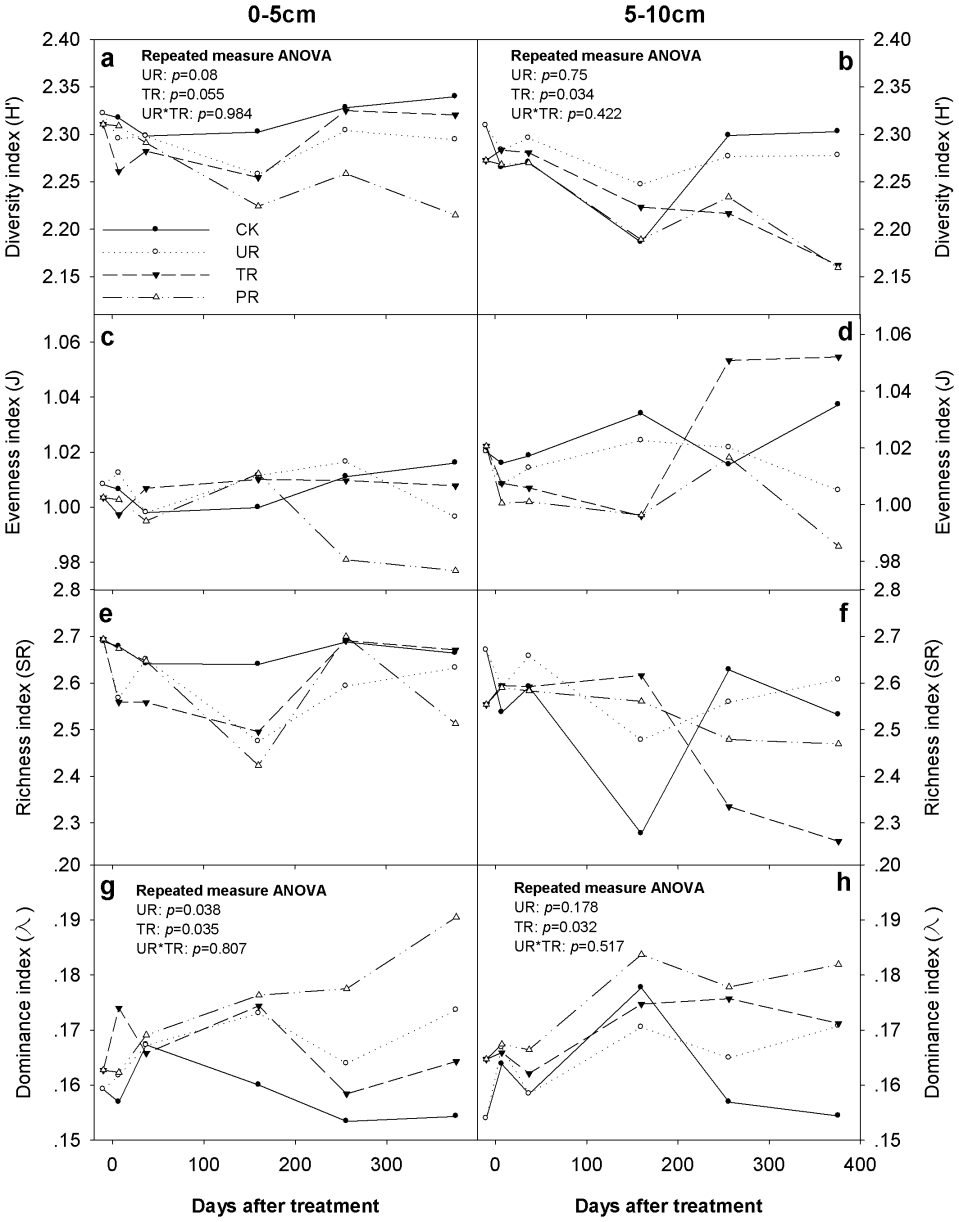
Microbial PLFA diversity as affected by vegetation removal during the study. Shannon–Wiener diversity index (*H′*), Pielou evenness index (J), Margalef richness index (SR) and Simpson dominance index (λ) under control (CK), understory removal (UR), tree removal (TR), and all-plant removal (PR) in each sampling event at 0–5 cm (a, c, e, g) and 5–10 cm (b, d, f, h) soil depths.

There was a significant time effect on all the microbial variables, namely, Shannon–Wiener diversity index (*H′*), Pielou evenness index (J), Margalef richness index (SR), and Simpson dominance index (λ) (*p*<0.05) (Fig. 1). There were trends that understory removal and overstory removal decreased soil microbial PLFA diversity at 0–5 cm soil depth by 0.024 (*p* = 0.08) and 0.026 (*p* = 0.055) units, respectively (Fig. 1a). Overstoty removal significantly reduced soil microbial PLFA diversity at 5–10 cm soil depth by 0.048 units (*p* = 0.034) (Fig. 1b). Repeated measure ANOVA showed that understory removal and overstory removal apparently increased dominance index at 0–5 cm soil depth by 0.007 (*p* = 0.038) and 0.008 (*p* = 0.035) units, respectively (Fig. 1g); and overstory removal significantly increased dominance index at 5–10 cm soil depth by 0.011 units (*p* = 0.032) (Fig. 1h). There was no remarkable effect of vegetation removal on evenness index and richness index of microbial PLFA profile at both 0–5 cm and 5–10 cm soil depths during the study (Fig. 1c, d, e, and f). No interaction effect of understory removal and overstory removal on soil microbial variables was found during the study.

### Nematode Diversity

**Figure 2:**
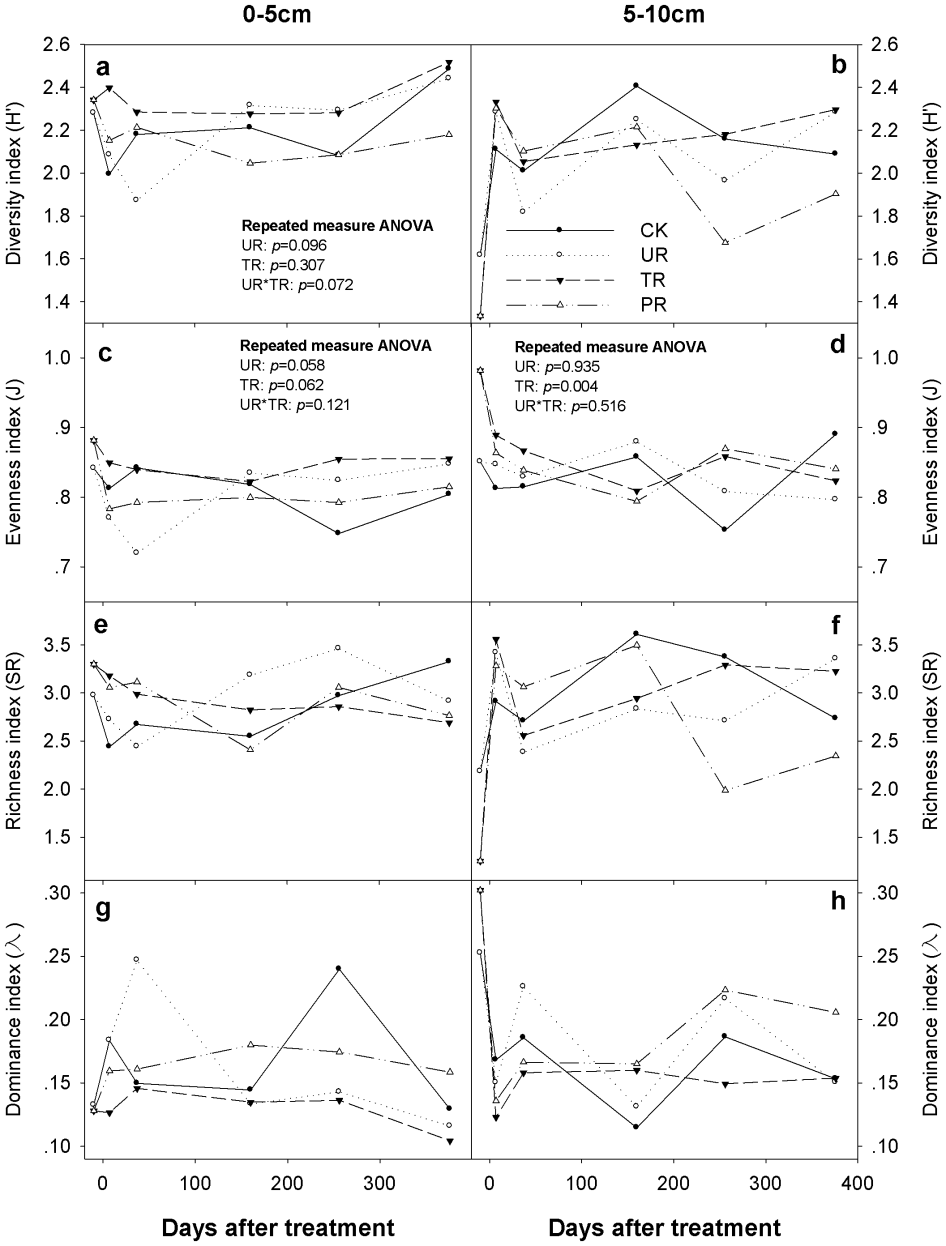
Nematode diversity as affected by vegetation removal during the study. Shannon–Wiener diversity index (*H′*), Pielou evenness index (J), Margalef richness index (SR) and Simpson dominance index (λ) under control (CK), understory removal (UR), tree removal (TR), and all-plant removal (PR) in each sampling event at 0–5 cm (a, c, e, g) and 5–10 cm (b, d, f, h) soil depths.

There was a significant time effect on all the nematode variables, namely, Shannon–Wiener diversity index (*H′*), Pielou evenness index (J), Margalef richness index (SR), and Simpson dominance index (λ) (*p*<0.05) (Fig. 2). There were trends that understory removal decreased soil nematode diversity index by 0.086 units (*p* = 0.096) (Fig. 2a) and evenness index by 0.022 units (*p* = 0.058) (Fig. 2c) at 0–5 cm soil depth, respectively. Overstory removal tended to increase the soil nematode evenness at 0–5 cm soil depth by 0.022 units (*p* = 0.062) (Fig. 2c), and significantly increased the soil nematode evenness at 5–10 cm soil depth by 0.036 units (*p* = 0.004) (Fig. 2d). There was a trend that interaction of understory removal and overstory removal tended to affect soil nematode diversity index at 0–5 cm soil depth during the study (*p* = 0.072) (Fig. 2a). Neither soil nematode Margalef richness index (SR) nor Simpson dominance index (λ) were significantly affected by understory removal or overstory removal at either 0–5 or 5–10 cm depth (Fig. 2e, f, g, and h).

### Correlations between Soil Biodiversity and Soil Microclimates

**Figure 3:**
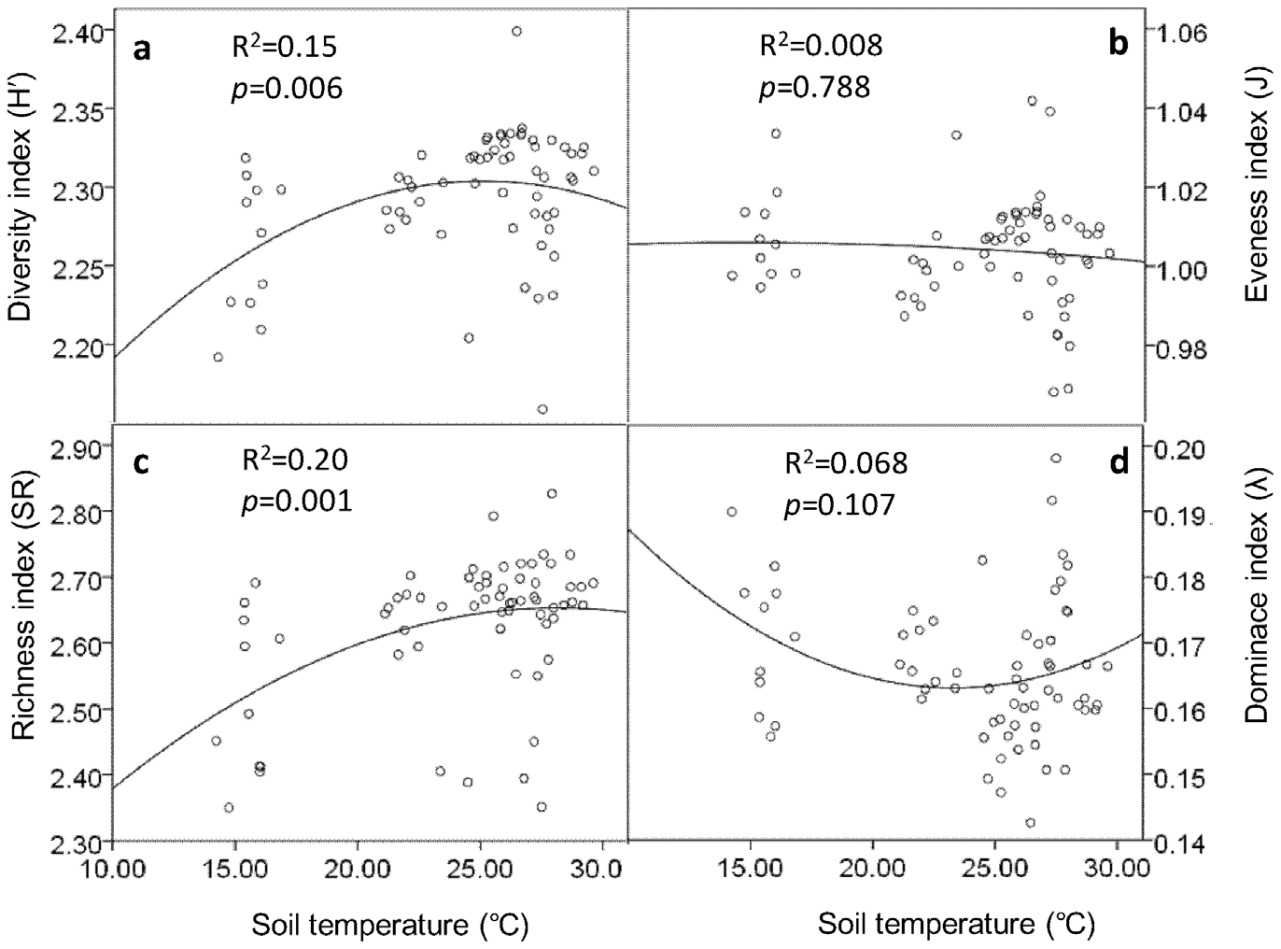
Relationships between indices of microbial PLFAs and soil temperature. Bivariate plots of the microbial PLFA diversity (a), evenness (b), richness (c) and dominance (d) indices versus soil temperature. Points represent results from all six sampling events at 0–5 cm soil depth. Soil temperatures used here were the mean daily temperatures of the six days that soil samplings were carried out.

**Figure 4:**
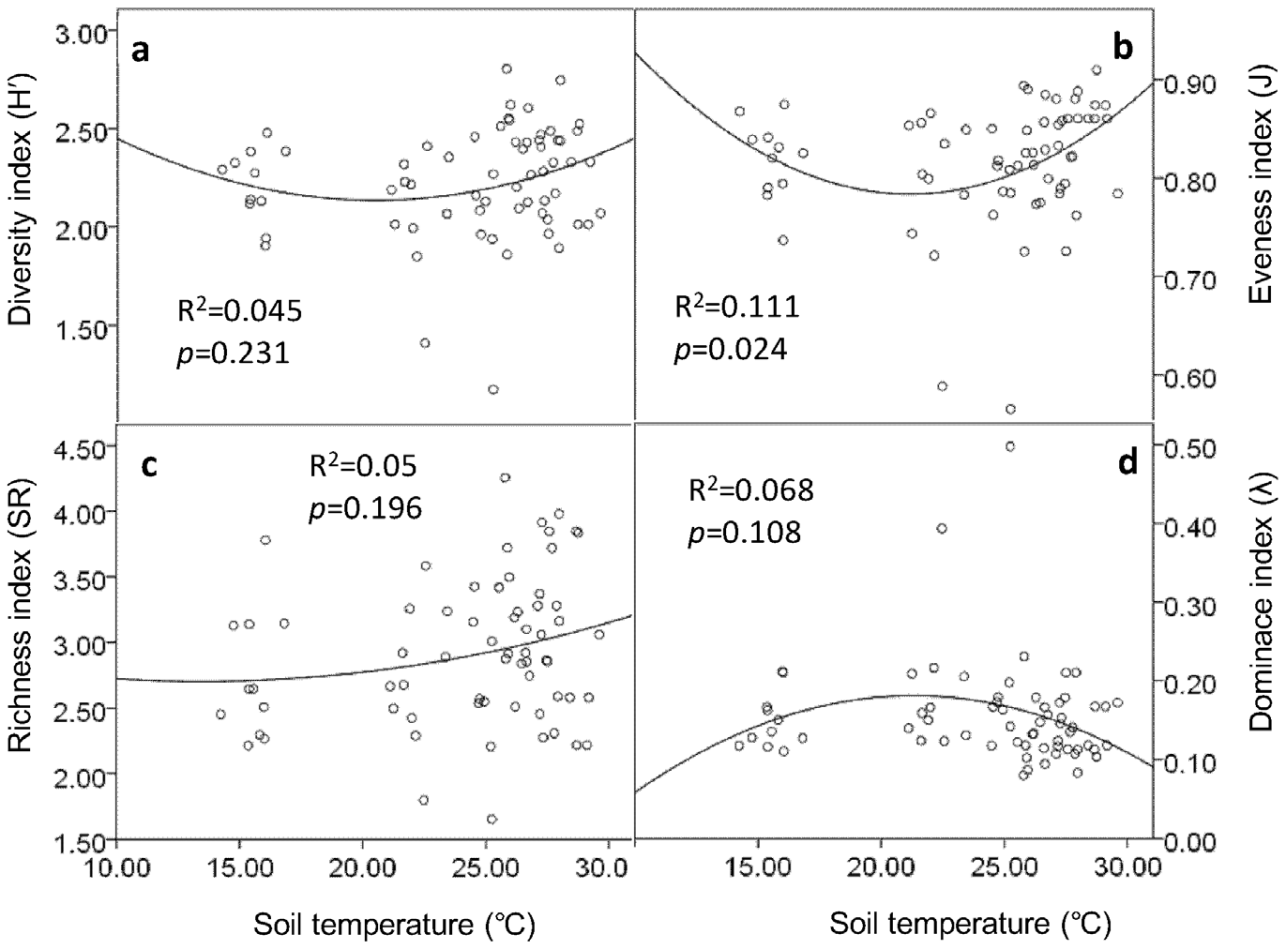
Relationships between indices of nematode diversity and soil temperature. Bivariate plots of the soil nematode diversity (a), evenness (b), richness (c) and dominance (d) indices versus soil temperature. Points represent results from all six sampling events at 0–5 cm soil depth. Soil temperatures used here were the mean daily temperatures of the six days that soil samplings were carried out.

Regression analysis showed that soil water content and soil biodiversity were not well fitting in either linear or quadratic model (Appendix S1). Coefficients of determination (R^2^) of both linear and quadratic regressions between soil water content and biodiversity indices (*H′*, J, SR, and λ) of soil microbial PLFAs and nematodes were very low (Appendix S1). Shannon–Wiener diversity index (*H′*) (R^2^ = 0.15, *p* = 0.006) and Margalef richness index (SR) (R^2^ = 0.02, *p* = 0.001) of microbial PLFA profile were quadratic correlated with soil temperature (Fig. 3a and c). Pielou evenness index (J) of nematode community was quadratic correlated with soil temperature (R^2^ = 0.111, *p* = 0.024) (Fig. 4b).

## Discussion

### Contributions of Understory or Overstory Vegetations to Soil Biodiversity

Apparently, all plant removal showed strongest suppression on soil microbial and nematode diversity in the present study. In addition, both understory removal and overstory removal reduced soil microbial PLFA and nematode diversities, which was inconsistent with our hypothesis that the effects of understory removal on soil biodiversity might be more powerful than overstory removal. Consistent with our finding, many studies reported that all plant removal had significant negative influence on soil biota in forest and grassland ecosystem [17], [31], [32]. Our previous study at the same site reported that understory removal had stronger negative effects on abundances and compositions of soil biota (microbes and nematodes) than overstory removal [20]. That was why our hypotheses stated as understory had greater contributions on soil biodiversity than overstory. Contrary to our hypotheses, our analyses clearly showed that the overstory vegetation impacted soil biodiversity too. In addition, overstory removal rather than understory removal significantly reduced amount of total PLFAs in 5–10 cm soil depth during the study (Appendix S2). There are many studies focused on the effects of overstory removal on soil biota because canopy tree harvest is a common forest management practice. For example, fifteen years after harvesting, diversity and structure of soil bacterial and fungal communities remained apparently altered by harvesting disturbances; and three fungi taxa (i.e. ectomycorrhizal fungi, saprobic taxa, and ascomycetes) and actinomycetes were most sensitive to harvesting disturbance in sub-boreal spruce forest in BC, Canada [15]. Both stem-only and whole-tree harvesting decreased microarthropod abundance in a temperate mixed conifer–hardwood stand [33] and in sub-boreal spruce stand [34]. However, these studies usually focused on size of populations and structure of communities of soil biota; there is no mention of how soil biodiversity responses to overstory removal in these studies. Furthermore, most of the studies did not clearly state whether understory was remained un-harvesting or not when overstory was harvested. In addition, understory vegetation is important ecological component that plays important roles in driving forest ecosystem functions and processes [20], [21]; however, it is usually overlooked in the previous studies. Several studies reported the relationship between understory vegetation and aboveground biodiversity (e.g., birds, reptiles, arthropods) [35], [36],[37]. Interactions between understory vegetation and soil biodiversity have not been documented yet. This is the first study reported the linkages between understory vegetation and soil microbial PLFA and nematode diversities.

Shannon–Wiener diversity index (*H′*) gives weight to rarer taxa, whereas Simpson dominance index (λ) gives weight to more abundant taxa [38], [39]. The suppressive effect of overstory removal on *H′* of soil microbial PLFA profile might indicate the linkage between overstory vegetation and soil rare microbial taxa. The promotive effect of overstory removal on λ of soil microbial PLFA profile might indicate the linkage between overstory vegetation and soil abundant microbial taxa. Therefore, overstory vegetation might link to both the rare and abundant taxa of soil microbial organisms. Although no significant effect of overstory removal on *H′* and λ of nematode community, overstory removal increased the Pielou evenness index (J) of nematode community, which implied that the abundance of nematode taxa did not differ from each other as much as their abundance before overstory removal. The likely reason might be that the rare species disappeared and were not collected after overstory removal, which eliminated the weight of the rare taxa to Pielou evenness index. In the similar way, understory vegetation linked to both abundant and rare taxa at 0–5 cm soil depth.

### Factor(s) that Affect Soil Biodiversity

Plant diversity may influence soil biodiversity via two main ways. High plant diversity may (1) enhance net primary productivity and (2) lead to high food resources (e.g. litter and root exudates) diversity, and subsequently supports high soil biodiversity [40]. However, many studies found plant species identities were more important than plant diversity in controlling soil biodiversity [41], [42], [43]; and sometimes the effect of plant identities can overrule the effect of plant diversity [41], [44], [45].

The bottom-up control might explain the alterations of soil biodiversity after vegetation removal [19], [20], [32]. *E*. *urophylla* is the only overstory species and *D. dichotoma* is the dominant understory species in the studied plantations. The productivity of both species are much high. The annual carbon increment of *E*. *urophylla* can reach 1960 gC m^−2^ yr^−1^ in the developmental stage in the studied area [25], while the biomass of *D*. *dichotoma* may contribute to 22%∼48% of total live biomass in *E. urophylla* plantations in the studied area (unpublished data). Both *E*. *urophylla* and *D*. *dichotoma* removal resulted in the reduction of carbon input to soil food web [19], which in turn may change soil biodiversity. The bottom-up control opinion was also supported by the differential responses of soil biodiversity at different depths to overstory and understory vegetation. Overstory removal decreased soil biodiversity at both 0–5 cm and 5–10 cm soil depths while understory removal mainly suppressed soil biodiversity at 0–5 cm soil layer. The reason might be that the overstory *E. urophylla* is a deep-rooted species and the dominant understory *D. dichotoma* is a shallow-rooted fern species with fibrous root on stolons. The deep-rooted species removal might reduce the availability of soil carbon and nutrients for soil biota at deeper soil depth. Previous studies reported that vegetation removal reduced fine root biomass in eucalyptus plantations in the studied area [19], [25]. Unfortunately, none of these studies differentiated either overstory or understory roots from the total plant roots and they did not report the composition of root at different soil depths.

In the present study, soil microclimate (especially soil temperature) explained a certain proportion of variability of soil biodiversity indices. Therefore, soil microclimate change that induced by vegetation removal probably contributed to the alteration of soil biodiversity. Supporting this hypothesis, growth temperature affected the cellular fatty acids composition of sulphate-reducing bacteria that were of marine origin [46]. In addition, our own data revealed that vegetation removal increased soil temperature and decreased soil water content in the later stage of the study, and the effect of understory removal on soil microclimate was greater than overstory removal.

The negative effects of vegetation removal on soil biodiversity also gradually increased over time, which indicated that alterations in bottom-up control and soil microclimate induced by vegetation removal were time-dependent. Firstly, vegetation removal significantly reduced the food resource for soil biota. However, the decomposition of the remained soil residuals (e.g., roots and litter) is time consuming. Therefore, the soil biodiversity declined with the exhaustion of soil residuals. Secondly, the soil microclimate changes that induced by vegetation removal were from March to October (mainly in wet season) in 2010, which was overlapped with the significant declines of soil biodiversity in this study. Thus, the seasonal variation of soil microclimate might be another reason for the time-dependent response of soil biodiversity to vegetation removal.

## Conclusions

Here we reported the first study designed to explore the relationship between overstory and understory vegetations and soil biodiversity. Our results showed that both overstory and understory vegetations correlated with soil biodiversity of Eucalyptus plantations in southern China. Interaction between overstory vegetation and soil biodiversity might be due to its high-biomass property that exerts a bottom-up control on soil biodiversity in the studied plantations. Interaction between understory vegetation and soil biodiversity might due to the mixing effect of vegetation identity and soil microclimate change after understory removal that drives soil biodiversity in the studied plantations. Eucalyptus has been extensively planted in many tropical and subtropical regions [25] and *Dicranopteris* is a common fern distributed through humid subtropical and tropical regions of the world [20]. Therefore, our findings might have great potential to extrapolate to large scales, especially to the humid subtropical and tropical region of the world.

## Supporting Information

Appendix S1 **Relationships between soil water content and soil biodiversity.**
(DOCX)

Appendix S2 **Amount of total soil PLFAs as affected by vegetation removal.**
(DOCX)
