# Supplementary material for: Contributions of Understory and/or Overstory Vegetations to Soil Microbial PLFA and Nematode Diversities in Eucalyptus Monocultures
Source: PLoS One. 2014 Jan 10;9(1):e85513. doi: 10.1371/journal.pone.0085513 (PMC3888421; doi:10.1371/journal.pone.0085513)
Supplement: Appendix S1 — Relationships between soil water content and soil biodiversity. (DOCX) [file pone.0085513.s001.docx]

Coefficients of determination (R^2^) with *F* and *p* values of linear and quadratic regressions between soil water content and biodiversity indices of soil microbial PLFAs and nematodes, respectively.

| Soil biota | Biodiversity indices | Linear model | |  |  | Quadratic model | |  |
| --- | --- | --- | --- | --- | --- | --- | --- | --- |
|  |  | R^2^ | *F* | *p* |  | R^2^ | *F* | *p* |
| Soil microbial | Shannon–Wiener diversity index (H′) | 0.028 | 4.032 | 0.047 |  | 0.059 | 4.406 | 0.014 |
| community | Pielou evenness index (J) | 0.040 | 5.811 | 0.017 |  | 0.041 | 2.968 | 0.055 |
|  | Margalef richness index (SR) | 0.003 | 0.429 | 0.514 |  | 0.020 | 1.427 | 0.243 |
|  | Simpson dominance index (λ) | 0.028 | 4.062 | 0.046 |  | 0.038 | 2.768 | 0.066 |
|  |  |  |  |  |  |  |  |  |
| Soil nematode | Shannon–Wiener diversity index (H′) | 0.012 | 1.675 | 0.198 |  | 0.020 | 1.455 | 0.237 |
| community | Pielou evenness index (J) | 0.039 | 5.744 | 0.018 |  | 0.040 | 2.910 | 0.058 |
|  | Margalef richness index (SR) | 0.004 | 0.525 | 0.470 |  | 0.004 | 0.261 | 0.771 |
|  | Simpson dominance index (λ) | 0.007 | 0.959 | 0.329 |  | 0.013 | 0.922 | 0.400 |
