# Supplementary material for: Contributions of Understory and/or Overstory Vegetations to Soil Microbial PLFA and Nematode Diversities in Eucalyptus Monocultures
Source: PLoS One. 2014 Jan 10;9(1):e85513. doi: 10.1371/journal.pone.0085513 (PMC3888421; doi:10.1371/journal.pone.0085513)
Supplement: Appendix S2 — Amount of total soil PLFAs as affected by vegetation removal. (DOCX) [file pone.0085513.s002.docx]

Amount of total soil PLFAs as affected by control (CK), understory removal (UR), tree removal (TR), and all-plant removal (PR) in each sampling event at 0-5cm (a) and 5-10cm (b) soil depths.
